# Exploratory behaviour towards novel objects is associated with enhanced learning in young horses

**DOI:** 10.1038/s41598-020-80833-w

**Published:** 2021-01-14

**Authors:** Janne Winther Christensen, Line Peerstrup Ahrendt, Jens Malmkvist, Christine Nicol

**Affiliations:** 1 Department of Animal Science, Aarhus University, Blichers Allé 20, 8830 Tjele, Denmark; 2 Royal Veterinary College, Hawkshead Ln, Brookmans Park, Hatfield, AL9 7TA UK

**Keywords:** Animal behaviour, Personality, Learning and memory, Operant learning

## Abstract

The mechanisms underlying individual variation in learning are key to understanding the development of cognitive abilities. In humans and primates, curiosity has been suggested as an important intrinsic factor that enhances learning, whereas in domesticated species research has primarily identified factors with a negative effect on cognitive abilities, such as stress and fearfulness. This study presents the first evidence of a link between object-directed curiosity and learning performance in young horses in two very different learning tasks (visual discrimination and pressure-release). We exposed young horses (n = 44) to standardised novel object tests at 5 months and 1 year of age and found consistency in responses. Standard indicators of fearfulness (e.g. heart rate and alertness) were unrelated to learning performance, whereas exploratory behaviour towards the novel objects correlated to performance in both learning tasks. Exploratory behaviour was unreinforced in the novel object tests and likely reflects the animal’s intrinsic motivation (i.e. curiosity), suggesting that this trait is favourable for learning performance. In addition to the insights that these results provide into cognition in a domesticated species, they also raise questions in relation to fostering of curiosity in animals and the impact that such manipulation may have on cognitive abilities.

## Introduction

The way cognitive functioning is affected by intrinsic and extrinsic factors is an important research area in behavioural and cognitive science. In humans and non-human primates, curiosity has been identified as a key intrinsic factor underpinning performance in cognitive tasks^1–4^. Curiosity can be defined as the motivation towards acquisition of novel information and is reflected in approaching and exploring novel stimuli where there is no immediate prospect of reward^3^. In orang-utans, curiosity—measured as exploratory behaviour towards novel stimuli—was a main predictor of problem-solving performance in a variety of tasks^3^. In farm animal species, however, research has primarily focused on the impact of restricted environmental conditions and other stressors related to the production environment, and how this may result in impaired cognitive abilities (e.g., in laying hens^5,6^, broilers^7^, dairy calves^8^, and pigs^9,10^). These effects may be mediated through stress hormones because prolonged high levels of glucocorticoids can negatively affect neurons within the hippocampus; a brain region central to learning and memory^11,12^. Acute stressors can also interfere during the learning phase if extrinsic stimuli in the environment cause shifts in attention away from the learning task^13^. Specifically, whereas low to moderate levels of arousal tend to facilitate learning, high levels of arousal generally impair it. Consequently, animals that exhibit high levels of fearfulness have been reported to display poorer learning and memory abilities^7,14^, as also demonstrated in domestic horses^15–18^. Furthermore, a growing body of evidence indicates a relationship between behavioural traits (also referred to as personality or temperament) and learning performance in a large range of animal species^19,20^.

Fearfulness is considered a relatively stable trait in domestic animals (e.g. in dogs^21,22^, mink^23^, cattle^24–27^, but see also Meagher et al.^28^). Boissy^29^ defined fearfulness as a basic psychological characteristic of the individual that predisposes it to react in a similar manner to a wide range of potentially frightening events. As prey animals, horses are innately neophobic and tend to avoid novel stimuli, and the most commonly applied fear test for horses is the novel object test where the animal is exposed to a novel stationary or suddenly moving object^30^. In contrast, the ‘open-field’ test—which is commonly used to test fearfulness and exploration in rodents—is not appropriate for horses as they do not avoid open areas and their responses in this type of test are difficult to interpret. Horse fearfulness is typically measured as behavioural responses such as alertness (i.e. elevated head and neck), avoidance of novel objects, latency to resume feeding after exposure to a sudden stimulus and physiological reactions such as heart rate; and these behavioural and physiological parameters usually show good correlations^15,31–33^. Thus, fearfulness can be reliably assessed in horses and appears to be a relatively stable trait across context and age^34–36^. Some studies have found fearfulness to be negatively related to exploratory behaviour. For example, farm animals raised in enriched environments were reported to be less fearful and to show more exploratory behaviour towards novel stimuli^37–39^. However, the relationship between fearfulness and exploratory tendencies may not be straightforward, as for example in rodents where these traits did not correlate and only the latter correlated to cognitive abilities^40,41^.

There is limited evidence that learning ability can be considered a single trait in animals due to variation in performance across different types of tasks^42^. While a few studies have revealed positive correlations in performance across different types of tasks, e.g. discrimination acquisition and reversal learning in song sparrows^43^ and bumblebees^44^ as well as across several tasks in a cognitive test battery in mice^40,41,45,46^, many other studies did not find such correlations (reviewed by Shaw and Schmelz^42^). Also in farm animals, a wide variety of learning tasks have been used to measure aspects of cognitive performance. The majority of learning tasks are based on positive reinforcement, i.e. the animal receives a reward such as food when it performs the correct behaviour (reviewed by Bushby et al.^47^). Typically, only one task, or two tasks within the same reinforcement regime, are used to test the effect of various extrinsic or intrinsic factors on cognitive performance. However, given the variation in performance across different tasks, studies should ideally include more than one type of task^42^. Furthermore, the animal’s motivation to obtain reinforcement can interfere with individual performance^48,49^. In horses, learning tasks based on negative reinforcement are particularly relevant because traditional horse training is based primarily on negative reinforcement, e.g. pressure from the rider’s legs, which is released when the horse performs the correct behaviour. Thus, domestic horses are ideal model animals for investigation of negative reinforcement learning^50^. Consequently, we included two learning tasks, targeting both positive (food) and negative (release of pressure) reinforcement regimes in our study. As the positively reinforced task, we used a simple, two-choice visual discrimination task, where the horses had to learn to differentiate between a rewarded and a non-rewarded container. As the negatively reinforced task, we used a recently developed pressure-release task^51^ where the horses had to learn to take a step to the side in response to pressure applied with an algometer to the horse’s hindquarters.

In this study, we infer fearfulness and curiosity from the horses’ responses to novel objects (fearful horses show increased alertness and heart rates; curious horses show novel object-directed exploratory behaviour). We aimed to investigate associations in fearfulness, curiosity and learning, which are of both fundamental and applied interest as these aspects influence horse welfare, performance and human safety. We measured young horses’ responses in standard novel object tests, pre- and post-weaning; at 5 months (1 test) and 1 year of age (2 tests) and determined the consistency of responses. Further, we investigated the potential association to learning performance in two learning tasks, targeting different motivations (positive and negative reinforcement). We predicted consistency of behavioural responses in novel object tests conducted after weaning, and a lower degree of consistency in responses between pre- and post-weaning tests. We further expected fearful animals to show reduced learning performance.

## Results

### Novel object tests at 5 months and 1 year of age

An overview of the horses’ responses in the Novel Object Tests is shown in Table 1. The 11 foals that touched the objects at 5 months did so for 2–25 s during the 120 s test. Thirteen horses touched the same objects for 2–23 s at 1 year of age. The 19 horses that touched the objects in the new object test (NOT2) did so for 2–19 s. Ten of the 11 foals that touched the objects at 5 months also touched the objects in both tests (n = 6) or in one test (n = 4) at one year of age, suggesting consistency in the tendency to approach and touch novel objects (McNemar’s test, p = 0.006). There were no differences between males and females on the four variables in the novel object tests at any age.

**Table 1:** Behaviour and heart rates (mean ± s.e.m. and median [25%; 75%]) in the Novel Object Tests at 5 months (NOT1_foal) and at 1 year of age (NOT2, and re-exposure to the NOT1 objects: NOT1_yearling).

Recorded variable	Age: 5 month N = 42	Age: 1 year N = 44	
	NOT1_foal	NOT2	NOT1_yearling
Alertness (s)	5.3 ± 1.5 2.0 [0; 7] N = 29	7.5 ± 1.8 3.0 [0, 9.0] N = 32	3.2 ± 1.0 2.0 [2; 4] N = 25
Exploration (touching objects, s)	2.5 ± 0.9 0 [0; 2] N = 11	2.2 ± 0.6 0 [0; 3] N = 19	1.7 ± 0.7 0 [0; 2] N = 13
Heart rate, avg (bpm)	70.4 ± 1.1 71.0 [67; 75] N = 40	76.8 ± 1.5 74.0 [71; 82] N = 41	76.5 ± 2.0 75.0 [69; 80] N = 41
Heart rate, max (bpm)	99.9 ± 3.3 97.5 [85; 110] N = 40	100.2 ± 2.2 97.0 [91; 108] N = 41	100.8 ± 3.7 94.0 [85, 115] N = 41

N denotes the number of horses showing the behaviour or with usable heart rate files. See “Methods” section for description of tests and recordings.

### Correlations within and between Novel Object Tests

In the 5 months test, the duration of alertness and exploration correlated negatively (r_s_ = − 0.42, p = 0.005) and both correlated to peak heart rates (alertness and max HR: r_s_ = 0.46, p = 0.003; exploration and max HR: r_s_ = − 0.31, p = 0.048). Similar within-test correlations were also present in NOT2 at 1-year: alertness and exploration (r_s_ = − 0.40, p = 0.008), alertness and HR (max HR: r_s_ = 0.48, p = 0.002; avg HR: r_s_ = 0.56, p < 0.001), exploration and max HR (r_s_ = − 0.31, p = 0.048), whereas in NOT1_yearling, the only significant correlations were between alertness and HR (max: r_s_ = 0.48, p = 0.002; avg: r_s_ = 0.59, p < 0.001).

Responses correlated significantly between the two 1-year tests, i.e. horses that showed alertness and had high heart rates in one test reacted the same way in the other test (HR avg: r_s_ = 0.86, p < 0.0001, HR max: r_s_ = 0.76, p < 0.0001; Alertness: r_s_ = 0.66, p < 0.0001). Likewise, the duration of exploratory behaviour correlated between the two 1-year test (r_s_ = 0.43, p = 0.004). Exploratory behaviour also correlated significantly between NOT1 at 5 months and at 1 year of age (r_s_ = 0.43, p = 0.005), whereas alertness and heart rates had slightly positive but insignificant correlation coefficients. The behavioural responses to unknown objects (NOT1_foal and NOT2 at 1-year) also correlated positively (Exploration: r_s_ = 0.38, p = 0.013 and Alertness: r_s_ = 0.34, p = 0.029), whereas heart rates did not, suggesting that heart rate responses to novelty may not be stable at 5 months of age, or that the mare’s presence in the opposite end of the test arena in the test at 5 months reduced heart rate responses in the most fearful foals (Difference in average heart rates in NOT1_foal and NOT 2 at 1 year: signed rank test: p = 0.016, and NOT1_foal and NOT1_yearling: paired t-test: p < 0.001).

### Learning tasks at 1 year of age

In the Negative Reinforcement task (NR), the horses had to learn to move away from pressure applied at an increasing rate on their hindquarter, i.e. take a step to the side with their hind leg^51^. The required pressure (median [25; 75%]) to move the horses across all 10 repetitions of the task was 15.4 [12; 22] Newton. A relatively large number of trials exceeded the actuator limit of 30 N (97 of 430, i.e. 23%). Performance was evaluated for each horse as the progression in required pressure during the 10 repetitions, i.e. the slope was calculated for each horse and used in the analysis. A negative slope may indicate that the horse learned to move away from pressure as less and less pressure was required (NR_slope, median for all horses: − 0.2 [− 1; 0.8]). Twenty-two horses had negative slopes (median: − 0.9 [− 1; − 0.5]), whereas 21 horses had positive slopes, indicating that these horses did not learn to yield to pressure within the 10 repetitions (median: 0.8 [0.3; 1]).

The majority of horses (n = 39 of 43) accomplished the Visual Discrimination task (VD). Horses with the highest learning performance made only five incorrect choices during the 10 container position switches (median incorrect choices for all 39 horses: 16 [13; 20]). The four horses that failed the test were assigned the maximum value of 55 incorrect choices (a random choice would result in 27.5 errors). Performance in the two learning tasks did not correlate, and there was no difference in performance between male and female horses.

### Associations between reactions to novel objects and learning performance at 1 year of age

**Figure 1:**
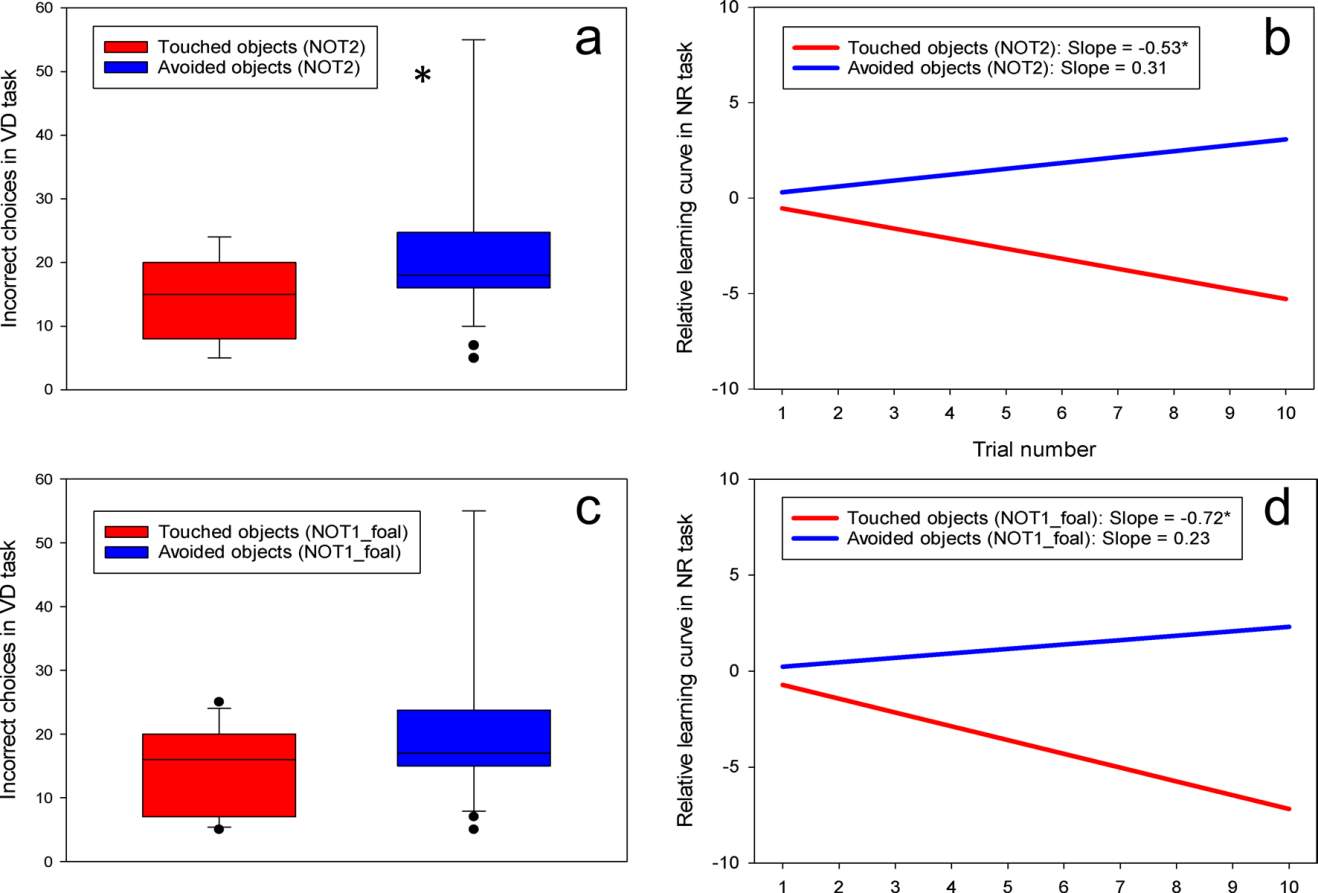
Learning performance of horses that either showed or did not show object-directed exploratory behaviour. (**a**) Horses that touched (red, n = 19) novel objects at 1-year (NOT2) made fewer incorrect container choices in the Visual Discrimination task (VD, p = 0.033), and (**b**) learnt more quickly in the Negative Reinforcement task (NR, p = 0.011). A negative slope indicates learning as the required pressure drops across the 10 trials. (**c**,**d**) A similar trend was observed already from the foal test at 5 months (NOT1) where foals that touched objects (red, n = 11) learnt more quickly in the NR test (p = 0.012), whereas the result did not reach significance in the VD test (p = 0.12). (**a**,**c**) Box shows median, interquartile range, lines show min and max and dots represent outliers. (**b**,**d**) Relative slopes; *indicates that the slope differs significantly from zero. Supplementary Figs. S1 and S2 online show plots of the raw data.

The standard indicators of fearfulness (heart rate and duration of alertness) did not correlate with performance in the two learning tasks. However, the duration of exploratory behaviour correlated significantly to learning performance: VD errors and NOT1_yearling: r_s_ = − 0.49, p = 0.001; VD errors and NOT2: r_s_ = − 0.35, p = 0.023, and NR slope and NOT2: r_s_ = − 0.38, p = 0.012. These correlations suggest that more exploratory horses had better learning performance. To investigate this potential relationship further, the 44 horses were divided into two groups: Horses that touched the novel objects and horses that did not touch the objects. These two groups of horses differed significantly in learning performance in both the VD and NR tasks for NOT2 (VD incorrect choices: Touching horses: 15 [8; 20] vs. Non-touching horses: 18 [16; 25], MWU-test: U = 140, p = 0.033, Fig. 1a; NR slope: Touching horses: − 0.53 ± 0.23 vs. Non-touching: 0.31 ± 0.21, t-test: t = − 2.7, p = 0.011; Fig. 1b) as well as for NOT1_yearling and VD (VD incorrect choices: Touching horses: 15 [7; 16] vs. Non-touching horses: 19 [16; 24], MWU: U = 77.5, p = 0.002), whereas the NOT1_yearling groups did not differ significantly in the NR task (NR slope: Touching horses: − 0.34 ± 0.36 vs. Non-touching horses: 0.06 ± 0.18, t-test: t = − 1.1, p = 0.27).

### Associations between reactions to novel objects at 5 months and learning performance at 1 year

There were no associations between alertness or heart rates in NOT1_foal and learning performance at 1 year, whereas the duration of exploratory behaviour tended to correlate to performance in the NR task (r_s_ = − 0.31, p = 0.052). Thus, to explore this relationship further, the horses were again analysed in two groups: Foals that touched the objects (n = 11) and foals that did not touch (n = 30) at 5 months. Touching foals had better performance in the NR task (NR slope: Touching foals: − 0.72 ± 0.31 vs. Non-touching: 0.23 ± 0.19, t-test: t = 2.62, p = 0.012, Fig. 1d), whereas VD performance did not differ significantly (VD errors: Touching foals: 14.09 ± 1.98 vs. Non-touching: 20.53 ± 2.35, t-test: t = 1.58, p = 0.12, Fig. 1c).

## Discussion

Novel object tests are frequently used to assess fearfulness in horses. Reactions towards novel, stationary stimuli reflect reactions in higher intensity fear tests (e.g. surprise tests^34^) and consistency between test situations and across age periods suggest that fearfulness can be considered a stable trait in horses^34,35^. Thus, the consistency in fear-related responses in the two novel object tests at 1 year of age was expected. Interestingly, behavioural parameters in the pre-weaning test at 5 months showed some consistency with behavioural parameters after weaning, despite the use of the dam as a motivator to pass around the objects in the foal test and food as a motivator in the 1-year test. Previously, Lansade et al.^34^ found consistency in horses’ behavioural responses over time, in particular licking and nibbling novel objects, in tests conducted at 8 months (after weaning) and at 2.5 years of age. Our results suggest that these behavioural responses may be observed as early as 5 months of age, even before weaning from the dam. Early-life assessment of behavioural traits can provide insight into the future usability of animals for various purposes and are important for optimizing handling and training techniques, and thereby increasing animal welfare and human safety. Thus, early assessment provide an interesting area of study into how consistent these traits are, and which life-events and timing of events that can influence the development of behavioural traits^19^.

The two learning tasks in our study were based on different reinforcement regimes, i.e. positive (learning to differentiate between two distinct containers to obtain food) and negative (learning to take a step to the side to remove pressure) reinforcement. The positively reinforced discrimination task is a widely applied cognitive test for farm animals (e.g.^52,53^) including adult horses which readily learn to discriminate between symbols and even faces in a two choice task^54^. This was also the case in the present study where most horses (91%) completed the task. The negatively reinforced pressure-release task is a recently developed task for horses, which aims to reflect practical training where horses are requested to yield to pressure applied somewhere on their body. The young horses in this study did not perform as well in the test as the Icelandic horses for which the test was originally developed^51^. In that study, the required pressure dropped significantly across the first 10 trials (repetitions), whereas the reactions of the young horses in this study were highly variable and only half of the horses had negative pressure slopes. This difference may be caused by a number of factors, e.g. that the horses in the current study were younger (1 vs. 3 years old in^51^) and less handled. In addition, 23% of the trials were censored because the pressure exceeded the actuator limit of 30 N. Thus, we speculate that more than 10 repetitions are required to obtain evidence of learning in all horses, in case of young relatively unhandled horses as in our study. There was no direct correlation in the horses’ performance in the two tasks, i.e. horses performing well in one task did not necessarily perform well in the other learning task, which is in accordance with previous studies^15,55^. Visser et al.^55^ used a negatively reinforced avoidance task—in which horses were exposed to air puffs if they did not move to another part of a test chamber upon hearing an auditory cue—and compared their performance to a food rewarded operant task. The authors reported that horses’ learning performance in the two tasks did not correlate, whereas there was some short- and long-term (14 and 30 months of age) consistency in performance within tasks. Individual horses may be differently motivated by positive and negative reinforcement: food motivation can contribute to performance in food rewarded tasks^48^, whereas pain/discomfort sensitivity potentially affects the motivation to respond to an aversive stimulus in negatively reinforced tasks. These individual differences in motivation can lead to variation in performance, even in animals with the same cognitive level. In our study, the poor performance of many horses in the NR task could further decrease the possibility of a correlation between the two learning tasks. Moreover, Wolff & Hausberger^56^ did not find correlations in learning performance in two food rewarded tasks (chest opening task and a detour task) suggesting that learning may be somewhat task-specific in horses. Alternatively, this could reflect that the applied tasks are of low ecological value for horses and thus performance in individual tasks may be more or less dependent on immediate intrinsic and extrinsic factors at the time of testing. For example, extrinsic stressors and emotional states have been reported to influence learning and cognitive flexibility in horses^15,17,18^. Further, Nawroth et al.^57^ suggested that performance in a visual discrimination task may be related to individual preferences for feature cues.

In contrast to our expectations, we found no association between traditional measures of fearfulness in horses and performance in the two learning tasks. Previous studies on adult horses reported a negative effect of fearfulness on performance in learning tasks under stressful conditions, which could be caused by fearful animals becoming more aroused and thus paying less attention to the task^15–18^. Our learning tasks were conducted separately and under low-stress conditions where a negative impact of fearfulness on learning performance may be less likely. However, horses that approached and manipulated novel objects at 1 year of age performed better in both learning tasks. For the negatively reinforced learning task, this trend was already apparent at 5 months of age. Thus, the tendency to show exploratory behaviour appear central to cognitive abilities in horses, as also shown in humans, non-human primates and rodents^1–4,40,41^. In rodent research, exploratory behaviour and fearfulness are traditionally assessed in the open-field test because rodents—unlike horses—tend to avoid open areas. Matzel et al.^40,41^ reported that fearfulness in the open-field test did not correlate to learning performance. These results suggest that exploration and fearfulness may not be a single dimension but rather reflect different aspects of temperament with varying importance for cognitive abilities. In accordance with previous results^31–33^, we found positive correlations between behavioural markers of fear (alertness) and heart rate variables within tests, whereas exploration showed weaker, negative correlations to heart rate in two of the three novel object tests. This may reflect that exploratory behaviour is not necessarily inversely related to fearfulness in young horses, possibly because tactile contact to novel objects may also lead to increased heart rates. It may be argued that the division of horses into two groups based upon whether they touched novel objects is a simplification. However, the occurrence of this behaviour is particularly interesting in animals that are typically neophobic. Actively seeking and making tactile contact to a novel object—despite the presence of food in the other end of the arena—is contradictory to the species-typical neophobic responses, and thus a potentially very interesting behaviour for further investigation.

In contrast to studies where exploratory behaviour could be beneficial for solving the learning task (e.g.^58^), the learning tasks in our study did not directly rely on exploratory behaviour during the task. So why did curious animals perform better? One explanation might be that curious individuals generally engage more with novel stimuli in their environment and the accumulated experience due to a history of curiosity could be expected to improve learning ability^3^. In our study, the young horses grew up in the same environment and individual differences in object-directed curiosity and learning may also reflect the early maternal environment^59,60^ as well as genetic effects. Another explanation might be that innate exploratory tendencies per se are correlated with an individual’s general learning abilities. Most knowledge on the impact of curiosity on cognitive abilities comes from studies on humans and non-human primates, and curiosity is considered a major factor for the evolution of intelligence and human culture. Yet, the biological function and mechanisms of curiosity remain poorly understood^1^, and the link between general exploratory tendencies and curiosity per se needs further study. Cross-species comparisons of how curiosity serves to motivate the acquisition of knowledge and learning could increase our understanding of the evolution and development of cognitive abilities. In domesticated species, where the human-animal relationship can be affected by cognitive processes^61,62^, fostering of curiosity in sensitive periods may be particularly relevant and provide new insights into the link between curiosity and cognition.

## Conclusion

Young horses’ intrinsic motivation to explore novel objects was positively associated to learning performance in both a positively and a negatively reinforced task, whereas traditional measures of fearfulness, such as alertness and heart rate responses, were unrelated to learning performance. These results are the first to suggest that novel object-directed curiosity could be central to cognitive performance in horses across different types of learning tasks; an association which has previously been demonstrated mainly in humans and primates. The result raises interesting questions in relation to fostering of curiosity and the impact of such manipulations on cognitive processes in domesticated animals.

## Material and methods

The study conformed with national legislation on animal experimentation (Danish Ministry of Justice, Act. no. 253 and §12 in Act. no. 1459), the ARRIVE guidelines, and the guidelines by the Ethical Committee of the International Society of Applied Ethology (https://www.applied-ethology.org/Ethical_Guidelines.html). Further, the study was granted ethical approval by the review board “Animal Welfare Body” at the Department of Animal Science, Aarhus University.

Forty-four domestic horses (Danish Warmblood foals; 16 females and 28 males) from one private stud were included with owner consent. The foals were all born at the stud (May–July) and kept together on large pastures with their mothers. The mares were only handled for insemination and occasional hoof treatment if required, and the foals were unhandled prior to the training for the experiment. During the autumn and winter, the mare-foal pairs were all housed together in a large barn with straw bedding and free access to pasture and to a roughage mixture. The foals were all weaned together in February (at 7–9 months of age) and were transported in groups by truck to a nearby facility belonging to the same stud, where they were housed in group boxes. Feeding and management was as before weaning.

### Novel object test at 5 months (NOT1_foal)

**Figure 2:**
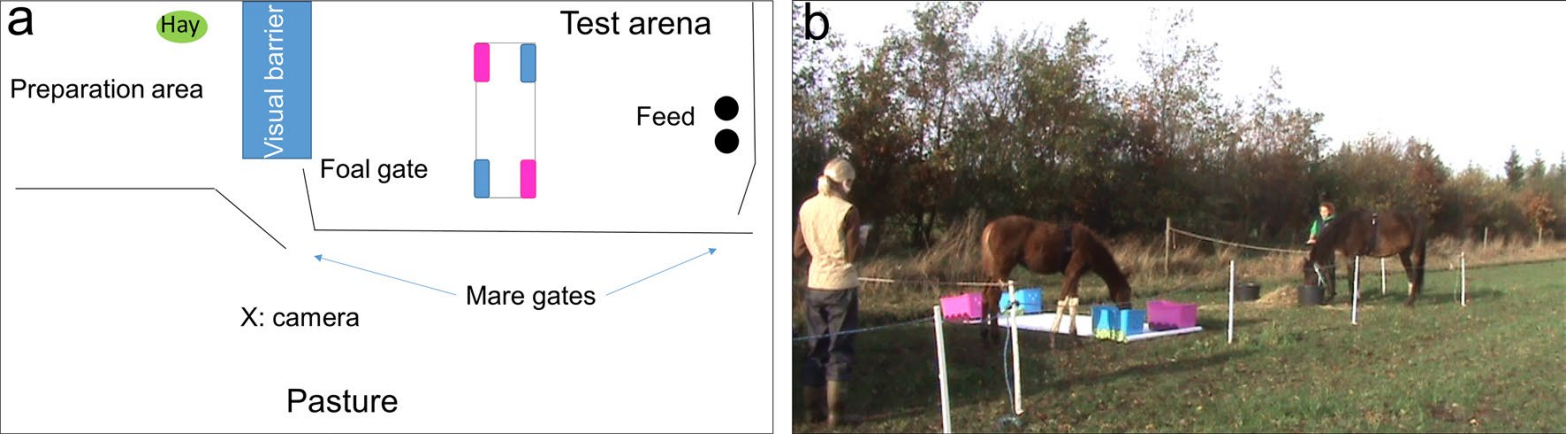
(**a**) Illustration of Novel Object Test (NOT1_foal) conducted with the 5 months old foals. A preparation and test arena were fenced off within the horses’ usual pasture. The foal was kept in the preparation area behind the visual barrier (2 m high) while the mare was led around the test arena and entered through the gate by the feed containers. The foal was gently pushed into the test arena and the 120 s test started when the foal placed a front leg in the arena. (**b**) Foal showing exploratory behaviour (touching and manipulating the objects).

A preparation area (4 × 5 m) and an adjacent test arena (5 × 10 m, Fig. 2) were created within the horses’ pasture. The other mares and foals in the herd were fenced off, preventing them from observing the test. When the foals were 4.5 months of age, the mare-foal pairs were habituated to the experimental set-up and the testing procedure, where the mare was caught and led with her foal to the preparation area. Using desensitization and combined reinforcement, the mares and foals were habituated to wearing an elastic girth with heart rate monitors and to voluntarily entering and feeding from feed containers in the test arena. To enable separate testing of the foals, we used the procedure for testing of young foals developed in Christensen^63^. The foal was habituated to staying behind a visual barrier in the preparation area (Fig. 2) while being groomed by the tail, which was a highly preferred spot for all foals. The mare was led around the test arena and entered from the opposite side where she was held and allowed to feed during the test. When the mare was in place, the foal was pushed gently into the test arena where it was free to move around and approach its mother. A foal was considered ready for testing when it calmly entered the test arena without resistance. This initial habituation training lasted two weeks, where the mare-foal pairs were trained 2–3 days per week for 15–20 min. For the test, novel objects (a sheet of white, shiny plastic (3 × 1.5 m) placed on the ground, with four plastic boxes, blue or purple 0.4 × 0.6 m, placed at each corner, see Fig. 2) were placed in the middle of the test arena. The 120 s test started when the foal placed a front leg in the arena. There was a 1 m gap between the objects and the fence on each side, allowing the foal to pass around the objects to reach its’ mother without touching the objects.

### Learning tasks at 1 year

**Figure 3:**
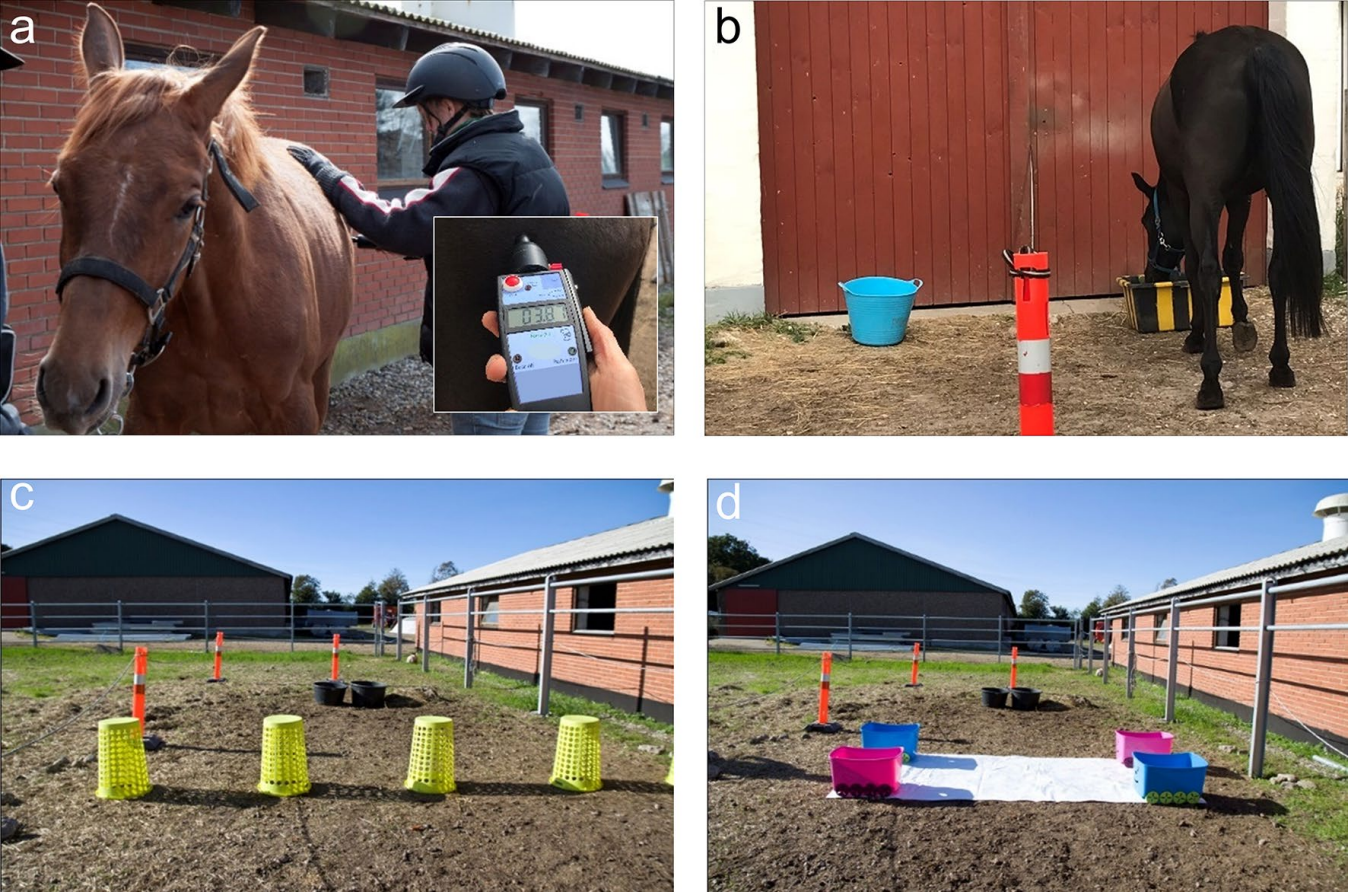
Learning and novel object tests conducted with the 1-year old horses. (**a**) Negative Reinforcement task (NR) in which pressure was applied with an algometer on the horse’s hindquarter at a pre-set increasing rate until the horse showed the correct response, i.e. moving a step to the side; (**b**) Visual Discrimination task (VD) where the horse should learn than only one of the two distinct containers contained food; (**c**) Novel Object Test 2 (NOT2); (**d**) Novel Object Test 1 (NOT1_yearling), which was similar to the 5 months test, except without the mare present.

The two learning tasks were conducted when the foals were 10–12 months old (hereafter termed horses). The horses had not been handled since the testing at 5 months. Thus, prior to testing they were habituated to halters and to be led to an outdoor testing arena (11 × 3 m, next to the stable), allowing visual contact to other horses through the open stable door. The horses were considered ready for testing when they could be led calmly around the test arena and willingly approached a food bucket when released. The foals required between 5 and 29 (median [25; 75% quartiles]: 10 [9; 15]) training sessions of approx. 10 min and were usually trained once per day. The horses were exposed to the two learning tasks on the same day with a 1 h break in between, during which the horse returned to the stable. The negative reinforcement task (NR) was a pressure-release task, where the horse had to learn to take a step laterally away from the pressure^51^. In each of 10 repetitions, pressure was applied manually with an algometer (TopCat Metrology Ltd., UK) on the left hindquarter of the horse (Fig. 3a). The force of pressure (Newton, N) was increased by 4 N/s until the correct response was obtained, after which the pressure was immediately removed to reward the horse. The algometer measured the force required to obtain the response. It was calibrated to 30 N and if more force was required to obtain the desired response, the experimenter used the other hand to apply the necessary force and the pressure was recorded as max (30 N). Learning performance was evaluated as the change in force across repetitions (NR_slope). A decreasing slope may suggest that the horse learned the task through negative reinforcement, as less and less force was required to obtain the response^51^.

In the visual discrimination task (VD), the horse had to learn that only one of two distinct containers (differing in size, shape, colour and pattern) contained food (Fig. 3b). The horses were randomly designated a correct (rewarded) container, and the task consisted of 10 container position switches (trials) following a predetermined pattern (position of the rewarded container: ‘l-r-l-r-l-l-r-r-l-r’ (n = 21) or ‘r-l-r-l-r-r-l-l-r-l’ (n = 22), with l = left and r = right). The horse was initially led to the correct container and allowed to eat a mouthful and then to the empty, incorrect container and allowed to sniff. During testing, the horse was released at the starting point and was free to approach a container. If the horse chose the correct container, it was allowed to eat a mouthful and moved on to the next trial. If it chose the incorrect container it was allowed to sniff the empty container after which it was led back to the starting point and allowed another attempt with the containers remaining in the same position. If a horse chose wrongly five times in a row, it was led to the correct container for a demonstration. Three such demonstrations were allowed. If the horse again chose wrongly five times in a row, it failed the task and was assigned 55 incorrect choices (the highest possible number of incorrect attempts). The task was chosen based on data suggesting that learning mainly occurred during the first session of the VD test (i.e. first 10 bucket position switches). Learning performance was evaluated as the total number of incorrect attempts during the task (i.e. lower values means better learning performance).

### Novel object tests at 1 year

These novel object tests were conducted after the learning tests when the horses were 12–14 months old, in a new test arena (along another stable wall, enabling the same arena size as during the 5 months test, 5 × 10 m and allowing only auditory contact with the other horses in the stable). The horses were again habituated to the test set-up prior to testing, incl. wearing elastic girths with heart rate monitors. A horse was considered ready for testing when it voluntarily entered the test arena on its own when released by the stable door, and walked directly to the feed containers placed opposite the entrance. For the tests, novel objects were placed in the middle of the arena (Fig. 3c,d) and the horse had to pass around these objects to go to the feed containers (120 s test duration). The first test included objects that the horses had not seen before (NOT2), whereas the second test was similar to the 5 months test (however, without the presence of the mare; NOT1_yearling). The two novel object tests were conducted on the same day with a habituation session (exposure to test arena without objects, 120 s) between the tests. The tests were conducted in the same order, since we were interested in test correlations, rather than comparing the relative intensity of the tests.

### Recordings

In the novel objects tests, heart rate (HR) was measured using Polar Equine RS800CX (RR recordings), consisting of two sensors, a W.I.N.D. transmitter and a wristwatch receiver. Water and gel were used to optimise contact between electrodes and skin. Data were downloaded using the software Polar ProTrainer, Equine Edtition 5 (Polar Electro OY, Kempele, Finland) and artefacts were corrected using the error correction function in this program. The average (HR avg) and maximum (HR max) heart rate from each 120 s test was extracted. Heart rate variability (HRV) was not used due to the short test duration (2 min), which is unsuitable for these parameters.

Behavioural responses, i.e. the duration (sec) of alertness (horse standing or moving vigilant, i.e. with elevated head and neck, while focussing on the objects) and of object exploration (horse makes physical contact—with muzzle, teeth or hoof—to the objects; includes manipulation of and moving the objects) were recorded from video recordings. In the NR learning task, the applied pressure (Newton) was noted in each trial and the learning curve (i.e. slope across the ten trials) was subsequently calculated and used as the performance parameter from this test. Negative slopes indicate that the task is learned as less and less pressure is required. In the VD task, (in)correct choices were noted for each position switch of the rewarded container and the total number of incorrect choices during the 10 switches was calculated and used as the performance parameter from this test, i.e. lower numbers indicate a good learning performance in this task. Two different staff groups conducted the learning tasks and the novel object tests incl. recording of behaviour from the videos; thus these staff groups were blind to the horses’ performance in the other tests.

Forty-four horses were included in the NOTs at 1-year and behavioural data were obtained from all, whereas HR files were lost from three horses due to poor conductance, i.e. n = 41 for HR data. Forty-two of the horses had also been tested at 5 months (Behavioural data: n = 42, HR data: n = 40). Forty-three horses were tested in the learning tests (one of the 44 horses was omitted due to dangerous behaviour (kicking out) towards the handler). Thus, for the analysis of NOT1_foal reactions and learning at 1-year: n = 41.

### Data analysis

Correlations within and between tests were analysed using Spearman’s rank correlations. The effect of age on reactions in the novel object tests was analysed using the paired t-test, or the non-parametric signed rank test in case the data did not meet the assumptions for the paired t-test; normal distribution (Shapiro–Wilk) and variance homogeneity (Brown-Forsythe). McNemar’s test was used to test the consistency of behaviour in the tests at 5 months and 1 year. Sex differences and differences in learning performance between the two groups of horses that either touched or did not touch the novel objects were analysed using the t-test, or the non-parametric Mann–Whitney U test. Linear regression was used to test whether slopes in the NR test differed from zero. All analyses were performed in SigmaPlot 14.0 (Systat Software Inc., San Jose, US). Results are presented as means ± se or medians [25;75% quartiles].

## Supplementary Information

Supplementary Figures.

Supplementary Table.

## Data Availability

Data is available as Supplementary information (Supplementary Table S3 online).
