# Supplementary material for: Exploratory behaviour towards novel objects is associated with enhanced learning in young horses
Source: Sci Rep. 2021 Jan 14;11:1428. doi: 10.1038/s41598-020-80833-w (PMC7809405; doi:10.1038/s41598-020-80833-w)

Supplementary Figures S1 and S2

Exploratory behaviour towards novel objects is associated with enhanced learning in young horses

Janne Winther Christensen[1,*], Line Peerstrup Ahrendt[1], Jens Malmkvist[1], Christine Nicol[2]

[1]Dept. of Animal Science, Aarhus University, Blichers Allé 20, 8830 Tjele, Denmark

[2]Royal Veterinary College, Hawkshead Ln, Brookmans Park, Hatfield AL9 7TA, UK

*Corresponding author: Email: jwc@anis.au.dk; Tel: +45 87158075

Supplementary Figure S1. Plots of raw data from the Visual Discrimination test (VD). (a) Total number of incorrect choices across 10 VD trials for the two groups of horses that either touched or did not touch the objects in the novel object test at 1 yr (each horse is illustrated by a symbol). Failed horses were assigned a value of 55 (Touched: n=1, Avoided: n=3). (b) Shows the same data, but with groups based on touching of objects in the foal test at 5 months.

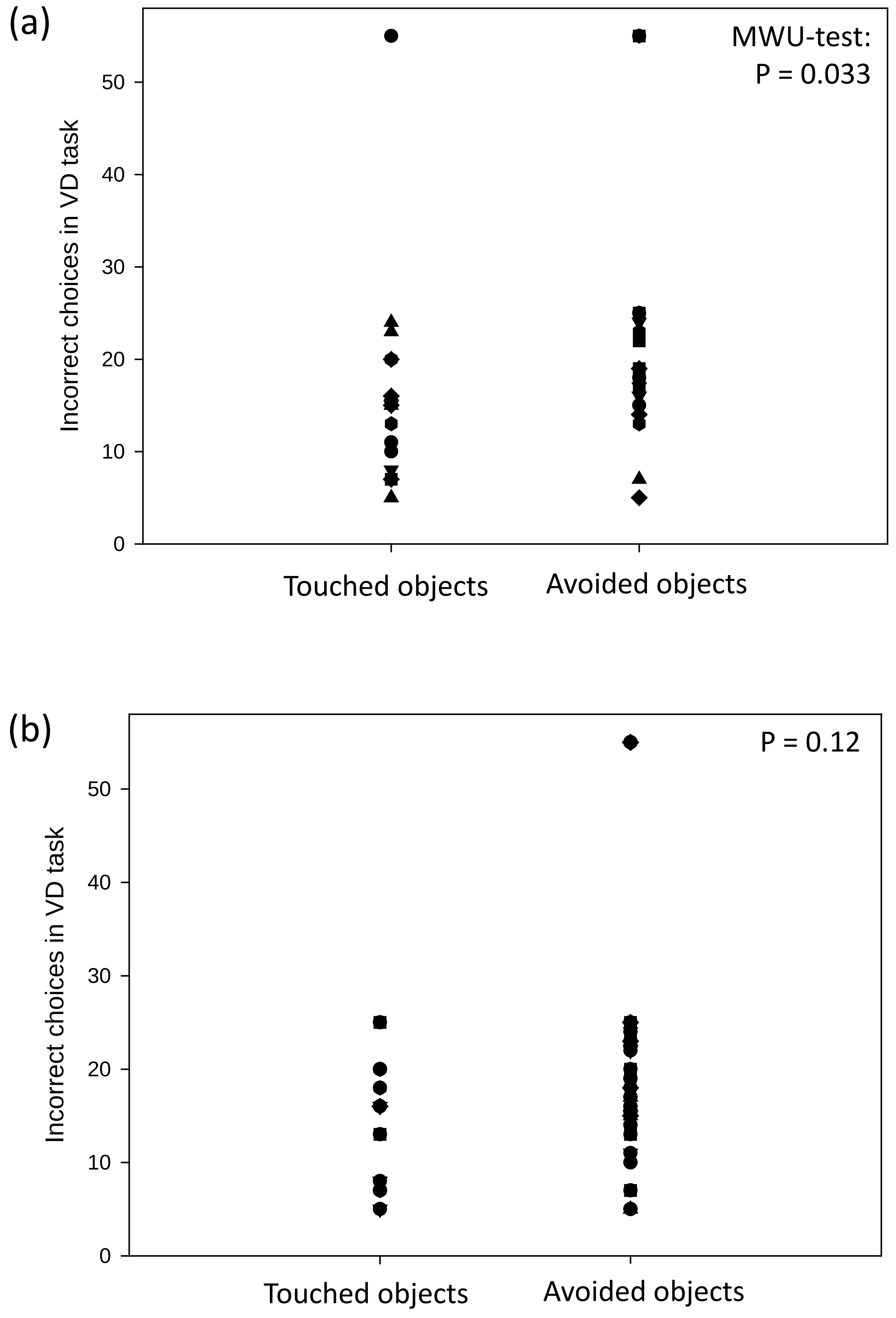

Supplementary Figure S2. Plots of raw data from the Negative Reinforcement test (NR). The level of pressure (N) applied with an algometer to each horse's hindquater to make it take one step to the side, in 10 subsequent trials (horse ID: Fxx). The slope for each horse was calculated and used in the analysis as a measure of performance. (a) Shows data for horses that touched the objects in a novel object test at 1 yr of age; (b) Shows data for horses that avoided the objects; (c) and (d) shows the same data, but with groups based on whether the horses had touched or avoided objects in the foal test at 5 months of age.

(a) NR test results for 1 yr horses that touched the objects

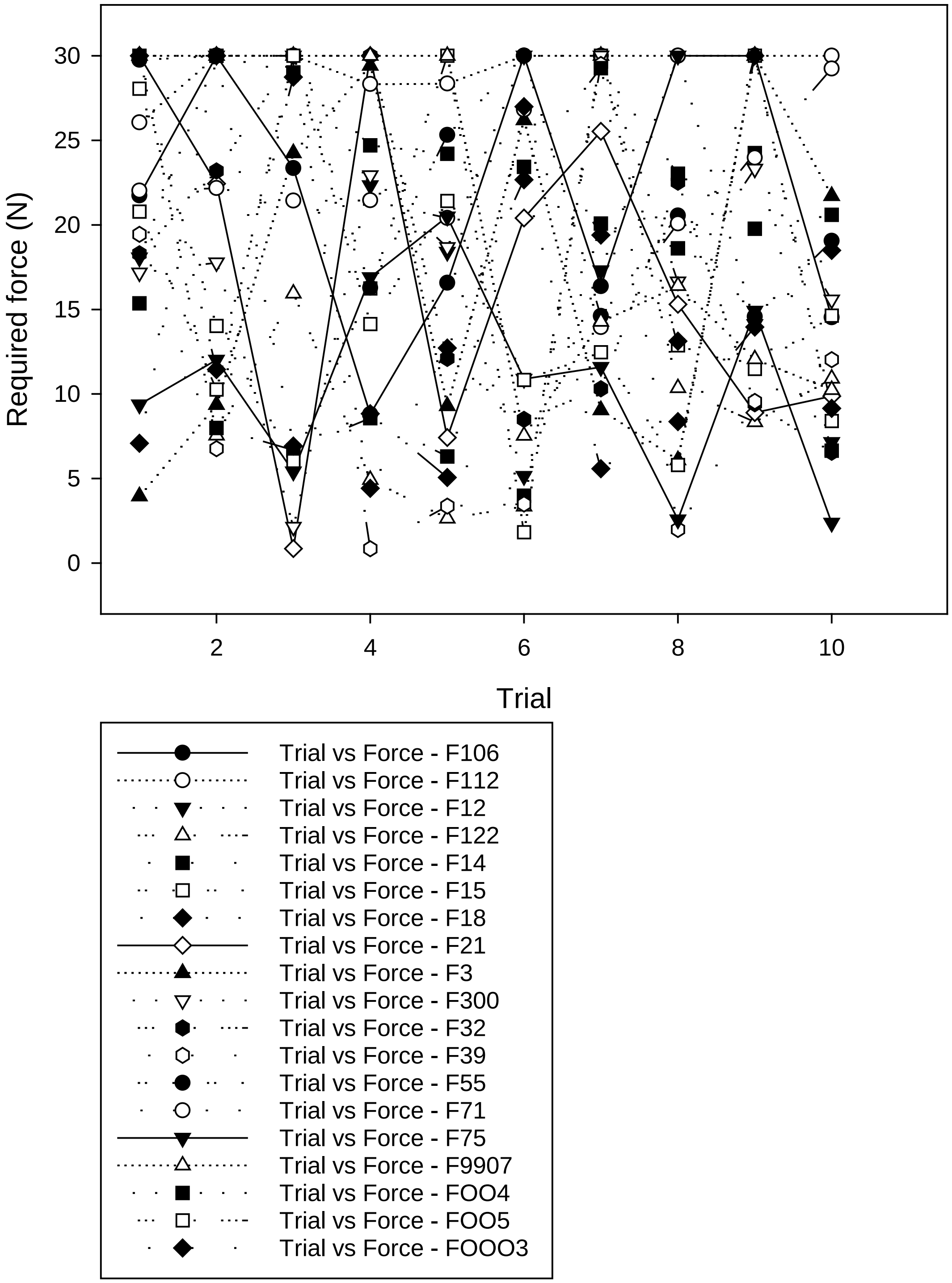

(b) NR test results for 1 yr horses that did not touch the objects

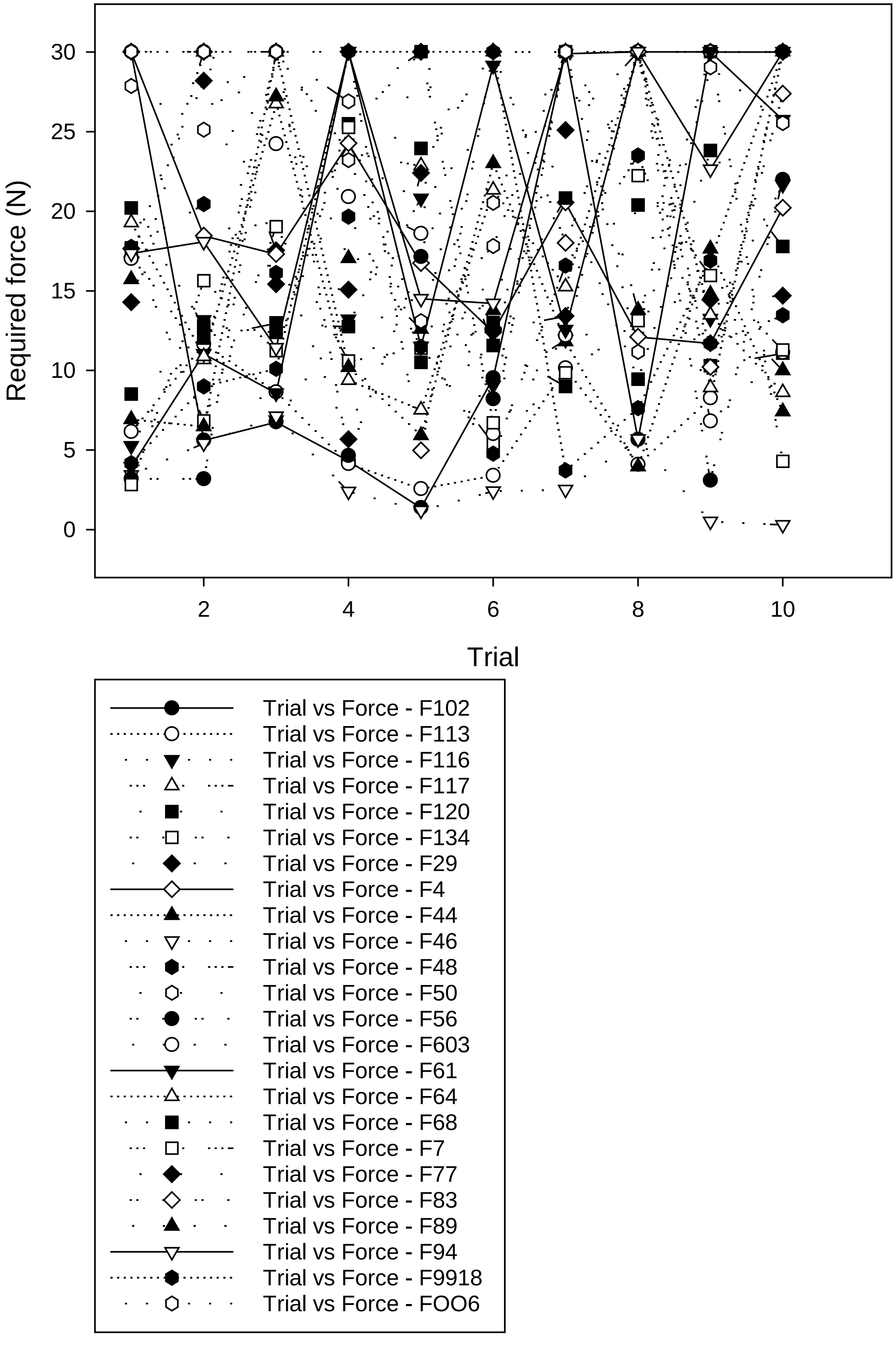

(c)

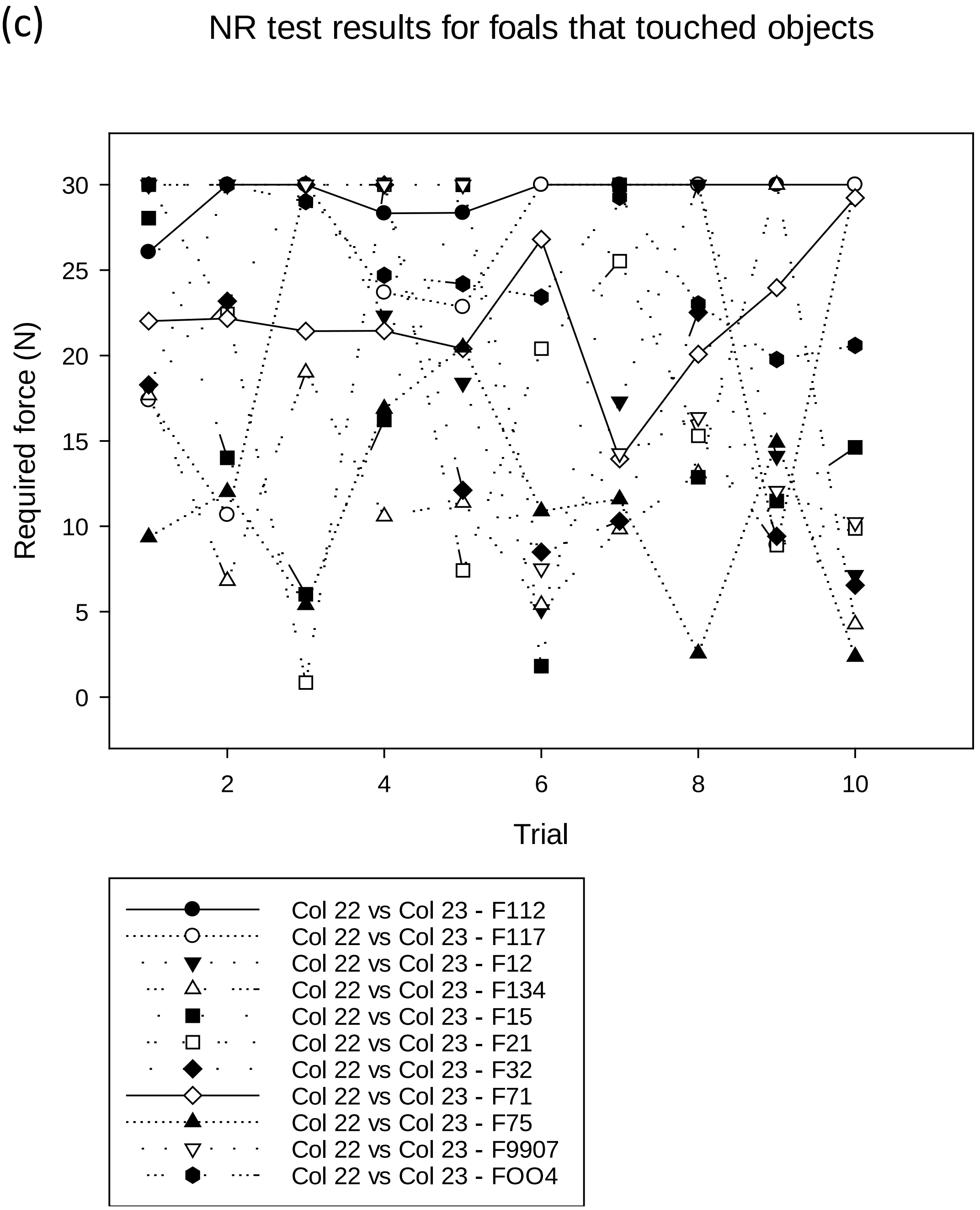
NR test results for foals that touched objects
Required force (N)
30
25
20
15
10
5
0
2
4
6
8
10
Trial
Col 22 vs Col 23 - F112
Col 22 vs Col 23 - F117
Col 22 vs Col 23 - F12
Col 22 vs Col 23 - F134
Col 22 vs Col 23 - F15
Col 22 vs Col 23 - F21
Col 22 vs Col 23 - F32
Col 22 vs Col 23 - F71
Col 22 vs Col 23 - F75
Col 22 vs Col 23 - F9907
Col 22 vs Col 23 - FOO4

(d) NR test results for foals that did not touch objects

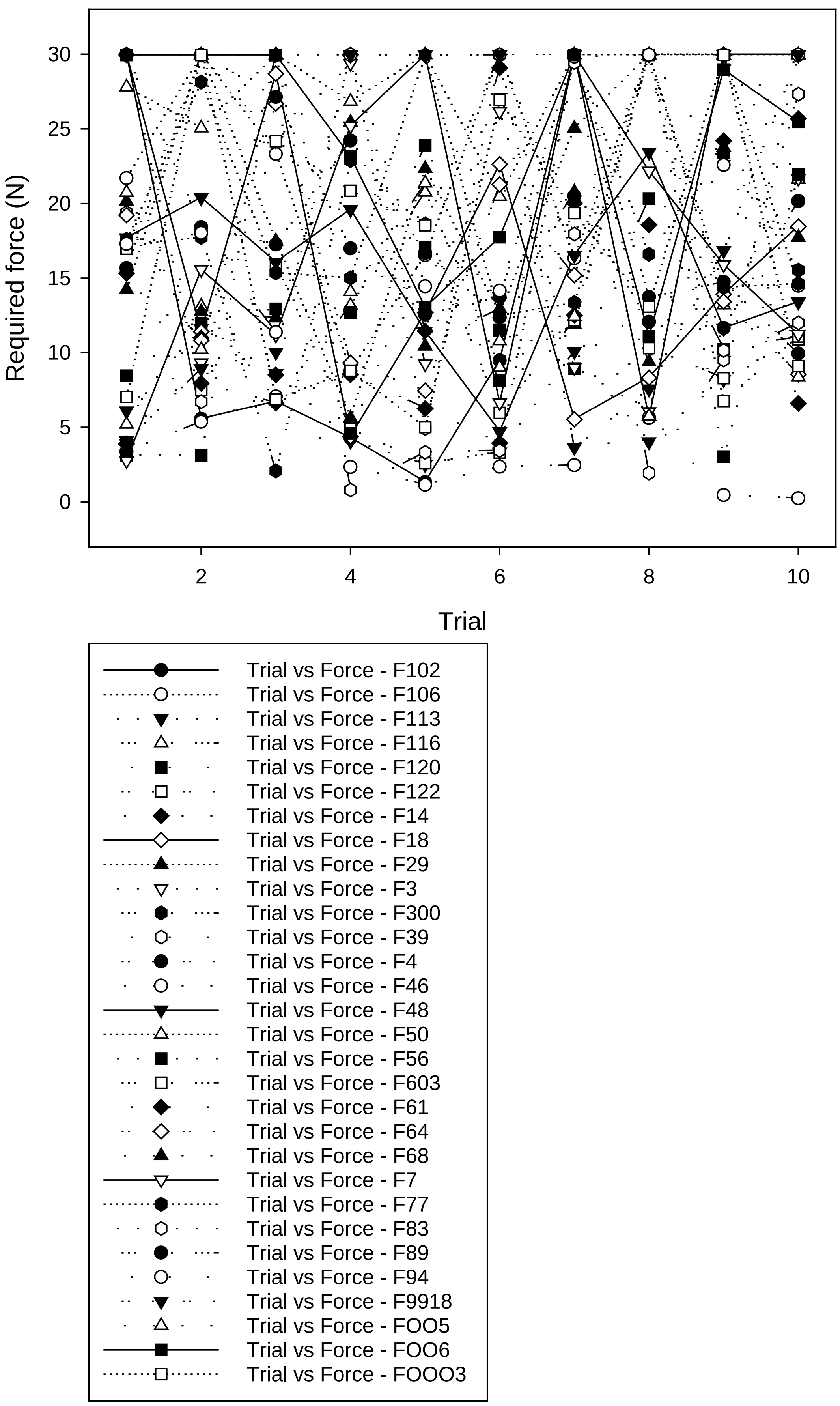

Supplement: Supplementary file 1 — Supplementary Figures. [file 41598_2020_80833_MOESM1_ESM.pdf]
